# Retraction Note to: Ultrasound Elastography supplement assessing nodal status of magnetic resonance imaging staged cervical N0 patients with nasopharyngeal carcinoma

**DOI:** 10.1186/s40644-019-0242-4

**Published:** 2019-08-02

**Authors:** Jian Li, Fei Han, Yunxian Mo, Xindan Chen, Yong Li, Feifei Zuo

**Affiliations:** 1 Department of Diagnostic and Interventional Ultrasound, State Key Laboratory of Oncology in South China, Collaborative Innovation Center for Cancer Medicine, Sun Yat-Sen University Cancer Center, No.651, Dong-feng-dong Road, Guangzhou, 510060 China; 2 Department of Radiation Oncology, State Key Laboratory of Oncology in South China, Collaborative Innovation Center for Cancer Medicine, Sun Yat-Sen University Cancer Center, No.651, Dong-feng-dong Road, Guangzhou, 510060 China; 3 Department of Radiology, State Key Laboratory of Oncology in South China, Collaborative Innovation Center for Cancer Medicine, Sun Yat-Sen University Cancer Center, No.651, Dong-feng-dong Road, Guangzhou, 510060 China; 4 Department of Pathology, State Key Laboratory of Oncology in South China, Collaborative Innovation Center for Cancer Medicine, Sun Yat-Sen University Cancer Center, No.651, Dong-feng-dong Road, Guangzhou, 510060 China

**Retraction Note to: Cancer Imaging**

**https://doi.org/10.1186/s40644-019-0199-3**

The Editors have retracted this article [1] because figure 2 has been substantially duplicated from a previously published article by Chen B et al., 2018 [2]. There is also significant and uncited overlap in the patient population between the two articles resulting in concerns relating to the scientific validity and novelty of the data.

None of the authors agree to this retraction.
